# Active Matrix Metalloprotease-9 Is Associated with the Collagen Capsule Surrounding the *Madurella mycetomatis* Grain in Mycetoma

**DOI:** 10.1371/journal.pntd.0002754

**Published:** 2014-03-27

**Authors:** Kirsten Geneugelijk, Wendy Kloezen, Ahmed H. Fahal, Wendy W. J. van de Sande

**Affiliations:** 1 Erasmus MC, Department of Medical Microbiology and Infectious Diseases, Rotterdam, The Netherlands; 2 Mycetoma Research Centre, Soba University Hospital, University of Khartoum, Khartoum, Sudan; University of Tennessee, United States of America

## Abstract

*Madurella mycetomati*s is the main causative organism of eumycetoma, a persistent, progressive granulomatous infection. After subcutaneous inoculation *M. mycetomatis* organizes itself in grains inside a granuloma with excessive collagen accumulation surrounding it. This could be contributing to treatment failure towards currently used antifungal agents. Due to their pivotal role in tissue remodelling, matrix metalloproteinases-2 (MMP-2) and 9 (MMP-9) or tissue inhibitor of metalloproteinases (TIMP) might be involved in this process. Local MMP-2 and MMP-9 expression was assessed by immunohistochemistry while absolute serum levels of these enzymes were determined in mycetoma patients and healthy controls by performing ELISAs. The presence of active MMP was determined by gelatin zymography. We found that both MMP-2 and MMP-9 are expressed in the mycetoma lesion, but the absolute MMP-2, -9, and TIMP-1 serum levels did not significantly differ between patients and controls. However, active MMP-9 was found in sera of 36% of *M. mycetomatis* infected subjects, whereas this active form was absent in sera of controls (P<0.0001). *MMP-2*, *MMP-9*, and *TIMP-1* polymorphisms in mycetoma patients and healthy controls were determined through PCR-RFLP or sequencing. A higher T allele frequency in *TIMP-1* (+372) SNP was observed in male *M. mycetomatis* mycetoma patients compared to controls. The presence of active MMP-9 in mycetoma patients suggest that MMP-9 is activated or synthesized by inflammatory cells upon *M. mycetomatis* infection. Inhibiting MMP-9 activity with doxycycline could prevent collagen accumulation in mycetoma, which in its turn might make the fungus more accessible to antifungal agents.

## Author Summary

Eumycetoma, mainly caused by the fungus *Madurella mycetomatis*, is a chronic infection which, without treatment, results in deformation of the infected body part. Inside the body, the fungus organises itself in grains which are surrounded by collagen. This collagen could act as a natural barrier for antifungal agents. Since collagen modulation is regulated by matrix metalloproteinase-2 (MMP-2), MMP-9 and tissue inhibitors of metalloproteinases (TIMPs), these enzymes could play a role in the formation of the collagen capsule surrounding the fungal grain. Indeed, we demonstrated that MMPs were found surrounding the mycetoma grain and that measurable levels of both MMPs were found in serum of both mycetoma patients and healthy controls. Only in mycetoma patients the active form MMP-9 was found. The presence of active MMP-9 in the serum of mycetoma-patients was not the result of lower levels TIMP-1 but more likely from differences in allele frequencies in the *TIMP-1* gene. In conclusion, our results showed an increased MMP-9 activity in mycetoma patients. We hypothesize that inhibition of MMP-9 activity by doxycycline will result in breakdown of the collagen capsule surrounding the grain, which in turn will make the entrance of antifungal drugs into the grain easier.

## Introduction

*Madurella mycetomatis* is the most prevalent causative organism of eumycetoma, a persistent, progressive granulomatous infection involving subcutaneous tissues and bones [1]. Mycetoma lesions are characterized by subcutaneous masses, sinuses and fungal grains, which commonly progress under inappropriate treatment resulting in deformation and disabilities of infected body parts [1]. To treat eumycetoma, a combination of surgery and treatment with antifungal agents is required [2]. Treatment with the currently used antifungal agents, ketoconazole and itraconazole, only facilitates surgical removal of mycetoma lesions as they induce encapsulation of the fungal grain with fibrous tissue [3], [4].

Encapsulation of the fungal grain by excessive collagen accumulation could be contributing to the *in vivo* observed treatment failure towards antifungal agents [1], [2], [5]. Collagen accumulation occurs due to a disrupted equilibrium of extracellular matrix (ECM) synthesis and degradation in which Matrix Metalloproteinases (MMPs) and Tissue Inhibitors of Matrix Metalloproteinases (TIMPs) play a pivotal role [6]. MMPs are classified into distinct groups according to their substrate specificity: collagenases (MMP-1, -8, -13), gelatinases (MMP-2, -9), stromelysins (MMP-3, -10, -11), matrilysin (MMP-7, -26), macrophage metalloestase (MMP-12), and membrane-type MMP (MMP-14 to MMP-25) [6]. MMP-2 and MMP-9 have the ability to degrade a variety of ECM constituents (e.g. gelatin, elastin, and various types of collagen) [6], [7]. Since both MMP-2 and MMP-9 are zymogens, proteolytic activation is prerequisite to become completely active [7]. Although it seems paradoxical, inhibition of MMP by a synthetic inhibitor decreased collagen accumulation in peritoneal sclerosis rats and bleomycin-induced pulmonary fibrotic rats [8], [9]. In addition, accumulation of collagen is correlated with MMP-2 or MMP-9 in several pathological conditions such as atherosclerosis [10], cardiac fibrosis in diabetic patients [11], and granulomatous fibrosis of rats with *Angiostrongylus cantonensis* infection [12], suggesting that collagen deposition can be promoted by gelatinases. Although the exact mechanism(s) explaining these observations have to be clarified, it is hypothesized that MMPs induce *de novo* ECM accumulation through its digestion of ECM constituents. Another explanation might be that MMPs provoke collagen accumulation via another pathway than ECM digestion.

Neither TIMP-1 nor MMP-2 and MMP-9 have been described to be involved in mycetoma pathogenesis. In this study, it is determined if MMP-2 and MMP-9 were expressed locally in the lesion by immunohistochemistry. Furthermore, MMP activity and absolute levels of MMP-2, MMP-9, and TIMP-1 in sera were assessed in both mycetoma patients and healthy endemic controls. In addition, polymorphisms in promoter regions of *MMP-2*, *MMP-9*, and *TIMP-1* were compared in both groups. The results obtained show that MMP-9 is associated with mycetoma.

## Materials and Methods

### Subjects

Genomic DNA of 125 *M. mycetomatis* infected patients from Sudan (72.8% male; 27.2% female) and 103 healthy endemic controls without *M. mycetomatis* infection (73.8% male; 26.2% female) were used for genotyping. Sera from another 44 male Sudanese *M. mycetomatis* mycetoma patients and 44 male healthy endemic controls were used to determine levels of MMP-2, MMP-9, and TIMP-1 and to determine gelatinolytic activity. Tissue sections from the foot were obtained in 1998 from Sudanese patients with *M. mycetomatis* infection. The patients' demographic characteristics were recorded and that included gender, duration of disease, lesion size and site of infection.

### Sirius red staining

To localize collagen fibres, tissue sections of 8 *M. mycetomatis* infected subjects were stained with Sirius red staining and subsequently photographed.

### Immunohistochemical staining

Immunohistochemical staining was used to assess whether MMP-2 and MMP-9 are expressed around the fungal grain. Deparaffinised tissue sections of the same 8 *M. mycetomatis* infected subjects used for the Sirius red staining were treated with 0.3% hydrogen peroxidase for 30 minutes to quench endogenous peroxidase activity. To inhibit aspecific binding of primary antibodies, specimens were incubated for 1 hour with 2% normal goat serum in PBST (0.05% Tween20 (Sigma, Zwijndrecht, The Netherlands) in PBS). Tissue sections were incubated overnight at 4°C with primary antibodies against MMP-2 (40 µg/ml; IM33 Calbiochem) and MMP-9 (40 µg/ml; IM09L Calbiochem). After 1 hour incubation with goat anti-mouse IgG HRP-conjugated antibody (1∶200; Dako, Heverlee, Belgium) at RT, immunoreactivity was visualized using 3-amino-9-ethyl-carbazole (AEC; Sigma, Zwijndrecht, The Netherlands). Mayer's hematoxylin (Sigma, Zwijndrecht, The Netherlands) was used for counterstaining. As a negative control, tissues were stained without the primary antibodies being used.

### ELISA

Absolute serum levels of MMP-2 and MMP-9 in serum of *M. mycetomatis* infected patients (n = 36) and healthy controls (n = 36) were determined utilizing Human MMP-2 and Human MMP-9 enzyme-linked immunosorbent assay (ELISA) kits (cat#: RAB0365, Sigma-Aldrich, Zwijndrecht, The Netherlands; and cat#: KHC3061, Invitrogen, Breda, The Netherlands). Human TIMP-1 ELISA kit (Cat#: OK-0163, Assay Biotechnology, Breda, The Netherlands) was used to assess the serum level of TIMP-1 in both study populations (n = 44 for both populations). Experiments were conducted according to the manufactures instructions.

### Gelatin zymography

Gelatinolytic activity in sera of *M. mycetomatis* infected patients and healthy endemic controls were determined by gelatin zymography. One µl serum was electrophoresed under non-reducing conditions on a 10% SDS-polyacrylamine gel co-polymerized with 1 mg/ml gelatin (Fluka Analytical, Zwijndrecht, The Netherlands). As a positive control 0.4 ng activated proenzyme MMP-2 and 0.1 ng activated proenzyme MMP-9 (Enzo Life Sciences, Antwerpen, Belgium) were used. After incubating the gel four times in 2.5% Triton X-100 (v/v) (Sigma, Zwijndrecht, The Netherlands) for 15 minutes, the gel was incubated in developing buffer (50 mM Tris (pH 7.5; Sigma, Zwijndrecht, The Netherlands), 200 mM NaCl (Merck, Amsterdam, The Netherlands), 5 mM CaCl2 (Merck, Amsterdam, The Netherlands) and 0.02% BRIJ35 (Calbiochem, San Diego, USA)) for 65 hours at 37°C. The gel was stained with 50% methanol (Fisher Scientific, Landsmeer, The Netherlands), 20% acetic acid (J.T. Baker, Deventer, The Netherlands), and 0.125% Coomassie Brilliant Blue R-250 (Sigma, Zwijndrecht, The Netherlands) and destained with destaining solution (30% methanol and 1% formic acid (J.T. Baker, Deventer, The Netherlands)) until transparent lysis bands were visible.

### Genotyping of MMP-2, -9, and TIMP-1 polymorphisms

Functional SNPs in promoter regions of *MMP-2* (−1306 C/T), *MMP-9* (−1562 C/T), and *TIMP-1* (+372 C/T), associated with altered transcriptional activity [13]–[15], were genotyped utilizing genomic DNA of 125 *M. mycetomatis* infected patients and 103 healthy controls. To determine *MMP-2* (−1306 C/T) genotype, DNA was isolated as described before [16], [17] and amplified using primers 5′-CTTCCTAGGCTGGTCCTTACTGA-3′ and 5′-CTGAGACCTGAAGAGCTAAAGAGCT-3′. The PCR reaction consisted of 40 cycles of 30 s denaturation at 94°C, 30 s annealing at 58°C and 30 s elongation at 72°C. The genotype of the resulting amplicon was determined by restriction fragment length polymorphism (PCR-RFLP) with *Sph*I. To determine the *MMP-9* (−1562 C/T) genotype, DNA was amplified using primers 5′-GCCTGGCACATAGTAGGCCC-3′ and 5′CTTCCTAGCCAGCCGGCATC-3′. The PCR reaction was similar to the one described for the *MMP-2* (−1306 C/T) polymorphism, only the annealing temperature was changed to 65°C. The genotype of the resulting amplicon was determined by restriction fragment length polymorphism (PCR-RFLP) with *Bfa*I. The *TIMP-1* (+372 C/T) genotype was identified by sequencing after amplification with primers 5′-GCACATCACTACCTGCAGTC-3′ and 5′-GAAACAAGCCCACGATTTAG-3′.

### Statistical analysis

Deviation from Hardy-Weinberg equilibrium for each polymorphism was calculated by the Pearson's χ^2^ test. Differences in categorical variables and continuous variables between *M. mycetomatis* infected patients and reference group were tested with Fisher's exact or Mann-Whitney test respectively. Statistical comparisons were carried out using GraphPad Prism 5.0 or GraphPad InStat 3.0 (GraphPad Software, San Diego California USA). P<0.05 was considered to be statistically significant.

### Ethics statement

Written informed consent was obtained from all participants and ethical clearance was obtained from Soba University Hospital Ethical Committee, Khartoum, Sudan.

## Results

### Excessive collagen deposition surrounds the fungal grain

**Figure 1:**
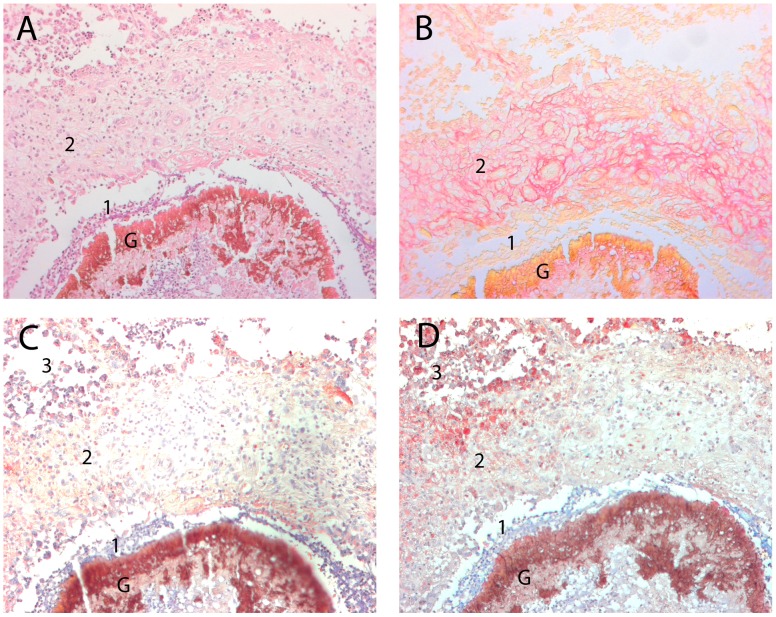
Collagen deposition and MMP-2 and MMP-9 expression around the *M. mycetomatis* grain. In this figure a representative picture of the *M. mycetomatis* grain insight the subcutaneous tissue from one particular patient is shown. In panel A, a HE staining is performed. The grain (G) and two different zones surrounding the grain are clearly visible (1,2). Zone 1, representing the neutrophil zone is relatively small around this typical grain. In zone 2, histocytes, capillaries, lymphocytes, plasma cells, fibroblasts and some macrophages are seen. In panel B, a Sirius red staining of the same area is shown. As is seen on this slide, collagen (coloured red) is mainly seen within zone 2. In panels C and D, MMP-2 and MMP-9 are detected by immunohistochemical staining, respectively. MMP-2 and MMP-9 positive cells are stained red, as a counterstaining hematoxylin is used. Relatively little MMP-2 positive cells were noted in the rim of zone 2, outside the layer where the collagen deposition was seen in panel B. MMP-9 staining was more heavily. Some cells in zone 2 stained positive for MMP-9, but more positive cells were seen at the rim of this zone (zone 3). No MMP-2 expression was noted in zone 1 surrounding the grain.

Collagen accumulation around the fungal grain was assessed by staining specimens of *M. mycetomatis* infected subjects by Sirius red. A representative photomicrograph of a Sirius red stained tissue section shows that the fungal grain is encapsulated with collagen deposition (Figure 1b). After this first collagen deposition ring, often a denser collagen capsule is seen at some distance of the grain. In that capsule typical collagen fibres are noted.

### MMP-2 and MMP-9 are expressed by immune-cells surrounding the fungal grain

In order to determine if the gelatinases MMP-2 and MMP-9 play a role in the encapsulation of the mycetoma grain, the presence of these two MMPs was demonstrated by immunohistochemical staining of tissue sections of patients infected with *M. mycetomatis* (Figures 1c and 1d). As is seen in figure 1c and 1d, both MMP-2 and MMP-9 were detectable as red cytoplasmatic staining in cells, mainly in zone 2 surrounding the grain. In the neutrophil zone (zone 1), little expression of either metalloproteases was noted, although in some patients also this zone showed expression of MMP-2 and MMP-9. Strikingly expression was mainly found in areas where little collagen deposition was seen. If there was heavy collagen deposition hardly any MMP-2 and MMP-9 expression was noted (figure 1). In slides where primary antibodies were omitted, coloration was absent (not shown).

### Active MMP-9 in serum is detectable in mycetoma patients but not in healthy endemic controls

**Figure 2:**
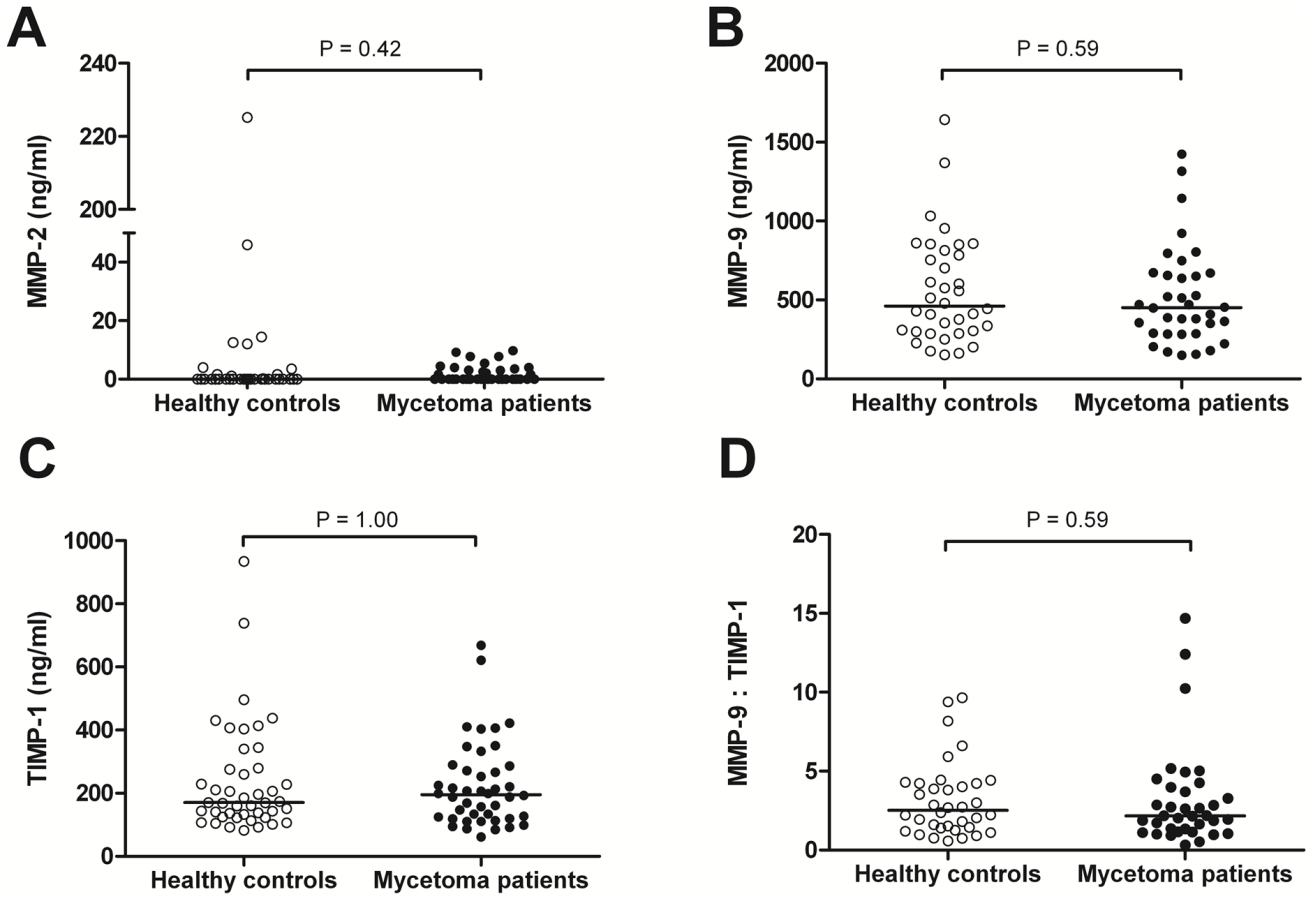
Concentrations of MMP-2 (A),MMP-9 (B), TIMP-1 (C), and MMP-9:TIMP-1 ratios (D) in sera of *M. mycetomatis* infected subjects and healthy endemic controls. Data is presented as median. Each dot represents the serum concentration or ratio of each (inhibitor of) metalloproteinases of one patient or healthy control. Significance was determined by Mann-Whitney test.

**Figure 3:**
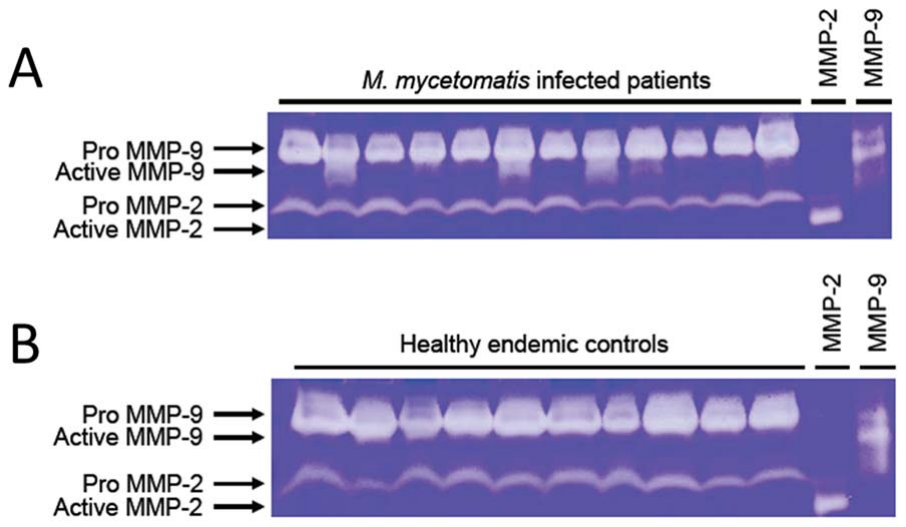
MMP activity in sera of *M. mycetomatis* infected patients and healthy endemic controls as demonstrated by gelatine zymography. One µl of serum of each subject was analyzed for MMP activity. Representative gelatine zymograms of 12 *M. mycetomatis* infected subjects (A) and 10 healthy endemic controls (B) are shown. The pro-active and active forms of MMP-2 and MMP-9 are marked with arrows. MMP-2: Activated proenzyme MMP-2; MMP-9: Activated proenzyme MMP-9.

In order to determine if the MMP-2 and MMP-9 expression was also found in serum, ELISAs were performed to determine the concentrations of MMP-2 and MMP-9 in sera of mycetoma patients and healthy controls. It appeared that MMP-2 was hardly detected in sera of either patients or controls (Figure 2A, MMP-2 median 0 ng/ml for both groups). There were no differences between the patients and the healthy controls (Mann-Whitney, p = 0.42). Also, similar concentrations of MMP-9 were found in sera of both *M. mycetomatis* infected patients and controls (Figure 2B, median concentration 451.6 ng/ml versus 461.2 ng/ml, respectively; Mann-Whitney, p = 0.57). The drawback by measuring MMP-2 and MMP-9 concentrations by ELISA is that it is not possible to distinguish between inactive and active MMP-2 and MMP-9. In order to distinguish between active and inactive gelatinase in sera of *M. mycetomatis* infected patients and healthy endemic controls, gelatin zymography was used. Characteristic gelatinolytic patterns due to the presence of pro-active and active forms of MMP-2 and MMP-9 in sera of *M. mycetomatis* infected patients and healthy controls are depicted in Figure 3. Active MMP-9 of 84 kDa was found in sera of 36% of *M. mycetomatis* infected subjects, whereas this active form was not present in sera of the control population (Fisher Exact, p<0.0001). No correlation was found between the presence of active MMP-9 and lesion size or disease duration (data not shown). Active MMP-2 (62 kDa) was absent in both groups. The pro-active forms of MMP-2 and MMP-9, 72 and 92 kDa respectively, were present in sera of all *M. mycetomatis* infected patients and healthy controls. Since mycetoma patients had more often active MMP-9 in their sera, while the total amount of MMP-9 (both active and inactive) did not differ, it was investigated if TIMP-1 levels differed between patients and healthy endemic controls by ELISA. This was done since TIMP-1 is known to block protease activity of both MMP-2 and MMP-9. It appeared that TIMP-1 serum levels of both groups did not significantly differ (Figure 2C, median 195.8 ng/ml for *M. mycetomatis* infected patients vs. 170.0 ng/ml for controls; Mann-Whitney, p = 0.99). Ratios of MMP-9 to TIMP-1 were comparable and did not reach statistical significance (Figure 2D; Mann-Whitney, p = 0.59).

### The T-allele of the TIMP-1 (+372 C/T) polymorphisms is associated with mycetoma in males

In order to determine if the difference in active MMP-9 levels was the result of genotypic differences between patients and healthy controls, we determined whether allele frequencies in functional polymorphisms in *MMP-2*, *MMP-9* and *TIMP-1* differed between *M. mycetomatis* infected patients and healthy controls by SNP analyses. All studied genotypes did not show deviation from Hardy-Weinberg equilibrium (p>0.05). Allele frequencies of *MMP-2*, *MMP-9*, and *TIMP-1* polymorphisms were compared between *M. mycetomatis* infected patients and healthy controls (Table 1). Since the *TIMP-1* gene is X-chromosome located, genotype analyses were stratified according to gender.

**Table 1:** Allele frequencies of *MMP-2*, *MMP-9*, and *TIMP-1* polymorphisms in *M. mycetomatis* infected patients and healthy endemic controls.

Polymorphism	Allele	Allele frequency in mycetoma patients (n = 125)	Allele frequency in healthy endemic controls (n = 103)	p-value for association (Fisher exact)
*MMP-2* (−1306 C/T)	C	235	198	0.39
	T	15	8	
*MMP-9* (−1562 C/T)	C	230	189	1.00
	T	20	17	
*TIMP-1* (+372 C/T) *males*	C	15	31	**0.0004**
	T	77	44	
*TIMP-1* (+372 C/T) *females*	C	18	12	0.53
	T	48	44	

The allele distributions for *MMP-2* (−1306 C/T) and *MMP-9* (−1562 C/T) polymorphisms did not significantly differ between *M. mycetomatis* infected subjects and controls (p = 0.39 and p = 1.00 respectively). The T allele frequency in *TIMP-1* (+372 C/T) polymorphism was significantly higher in male *M. mycetomatis* infected patients compared to the male reference group (46% versus 26%) (p = 0.0004). In female *M. mycetomatis* infected patients the allelic distribution in *TIMP-1*(+372 C/T) polymorphism did not significantly differ with female control subjects (p = 0.53).

## Discussion

Eradication of *M. mycetomatis* mycetoma remains challenging as *in vivo* treatment failure towards currently used antifungal agents is frequently observed. It has been reported that collagen accumulation contributes to limited penetration of chemotherapeutic agents into the granuloma [18], suggesting that a dense collagen network might influence drug accessibility. Therefore, diminished response upon antifungal treatment might be partly caused by excessive collagen accumulation in the mycetoma lesion. Unravelling the mechanism behind observed changes in tissue architecture around the fungal grain could direct to novel therapeutic options. In this study we investigated suitable candidates, MMP-2, MMP-9, and TIMP-1, as they are participants in ECM remodeling.

Both MMP-2 and MMP-9 were found to be expressed in the mycetoma lesion, and both were highly expressed locally surrounding the fungal grain. Constitutive expression of MMP-2 takes place in various cell types and is barely induced under pro-inflammatory conditions [19]. Furthermore, one of the characteristics of mycetoma is that during grain formation high amounts of neutrophils are recruited to the site of *M. mycetomatis* infection [17]. MMP-9 is constitutively expressed and stored in high quantities in granules of neutrophils, and several chemotactic chemokines and cytokines are able to induce degranulation of MMP-9 containing granules [20], [21]. Furthermore, inflammatory stimuli are able to upregulate MMP-9 expression in a wide range of inflammatory cell types, such as lymphocytes, monocytes, and neutrophils [19], [22].

Absolute MMP-2, MMP-9, and TIMP-1 serum levels were comparable between *M. mycetomatis* infected patients and healthy controls and did not reach statistical significance. However, since the ELISA measured both the pro-active and the active forms of MMP-2 and MMP-9, these observations have only a limited value. Therefore, MMP-2 and MMP-9 activity was tested by gelatin zymography. Despite comparable absolute serum levels in both groups, MMP-9 activity was significantly higher in the *M. mycetomatis* mycetoma population. A higher MMP-9 activation could be the result of a higher MMP-9 expression or a lower TIMP-1 expression. TIMP-1 inhibits MMP-9 activity by forming a 1∶1 stoichiometric non-covalent complex [6]. Disruption of MMP-9:TIMP-1 complexes result in release and activation of MMP-9. Several other participants in MMP-9 activation have been described, including protease-based activators (e.g. trypsin [23] and neutrophil-derived elastase [24]) and other MMPs [21]. Although we only found activated MMP-9 found in mycetoma patients, there was still a large proportion of the patients in which we did not find the activated form. Similar findings were reported for patients with severe sepsis [25]. Only in 10 out of 20 patients with severe sepsis on the intensive care unit, activated MMP-9 was found [25]. Again no correlation with disease severity was noted [25]. Why we measured in one patient active MMP-9 and the other not is not clear. Several reasons could be attributing. First of all, we only took one time-point and these time-points differ for each patient. MMP-9 expression could be dependent on the disease stadium, although we did not find a correlation with the disease duration or the size of the lesion, other factors might be responsible such as if the patient had at the time of sampling discharging sinuses or not. Furthermore we did not record if the patient had other infections. Furthermore, it is also plausible that co-infections could play are role since they are frequently reported in mycetoma [26] and a correlation between mycetoma and schistomiasis was also recently reported [27]. Differences in MMP-9 expression between the *M. mycetomatis* infected patients individually and between patients and healthy endemic controls as a group could also be caused by genetic differences. We therefore genotyped functional polymorphisms in the promoter regions of *MMP-2* (−1306 C/T), *MMP-9* (−1562 C/T), and *TIMP-1* (+372). While no significant difference in allele distributions in the *MMP-9* (−1572 C/T) polymorphism was found, other SNPs in the promoter region of *MMP-9* or *MMP-9* itself were not investigated and could contribute to increased MMP-9 activation in *M. mycetomatis* infected patients. In contrast, a genetic difference between both groups was found for *TIMP-1* (+372 SNP). The T allele frequency in *TIMP-1* SNP in male *M. mycetomatis* infected patients was significantly higher compared to healthy controls. In man, T allele associated transcriptional activity of *TIMP-1* is lower than C allele associated transcriptional activity [15], suggesting that TIMP-1 production and thereby MMP inhibition in these subjects is reduced. This finding might explain previously reported male predominance in mycetoma [1]. Due to lower T allele associated transcriptional activity of *TIMP-1*, we expected reduced TIMP-1 serum levels in *M. mycetomatis* infected subjects, but this was not the case. However, since mycetoma is a localized infection, a localized reduction of TIMP-1 could result in higher MMP-9 levels in the lesion, which, in its turn, could be found in serum.

In this study we showed that collagen is indeed encapsulating the grain and MMP-9 is the collagenase activated during *M. mycetomatis* infection. The question remains what the exact function of grain encapsulation is. Is this encapsulation beneficial to the host, by keeping the *M. mycetomatis* infection localized and preventing the spread of infection? Or prevents the collagen capsule surrounding the fungal grain the penetration of drugs into the grain? If the latter would be the case, one could consider adding the antimicrobial agent doxycycline to the currently used therapeutic strategy. Doxycycline is a potent MMP inhibitor which is able to reduce MMP-2 and MMP-9 mRNA expression and MMP-2 production *in vitro* and thereby attenuates collagen accumulation in pulmonary fibrosis [28]. By attenuating the collagen deposition around the grain, ketoconazole and itraconazole might be able to better penetrate to the fungus.

In summary, the results obtained in the present study show increased MMP-9 activity during *M. mycetomatis* infection, suggesting that MMP-9 is associated with *M. mycetomatis* mycetoma.

## References

[1] Ahmed AO, van Leeuwen W, Fahal A, van de Sande WWJ, Verbrugh H, et al (2004) Mycetoma caused by *Madurella mycetomatis*: a neglected infectious burden. Lancet Infect Dis 4: 566–574. 15336224 10.1016/S1473-3099(04)01131-4

[2] Ahmed A, van de Sande WWJ, Fahal A, Bakker-Woudenberg IA, Verbrugh H, et al (2007) Management of mycetoma: major challenge in tropical mycoses with limited international recognition. Curr Opin Infect Dis 20: 146–151. 17496572 10.1097/QCO.0b013e32803d38fe

[3] Fahal AH (2006) Mycetoma, Clinicopathological Monograph. Khartoum: Khartoum University Press.

[4] Fahal AH, Rahman IA, El-Hassan AM, Rahman ME, Zijlstra EE (2011) The safety and efficacy of itraconazole for the treatment of patients with eumycetoma due to *Madurella mycetomatis* . Trans R Soc Trop Med Hyg 105: 127–132. 21247608 10.1016/j.trstmh.2010.11.008

[5] van de Sande WW, de Kat J, Coppens J, Ahmed AO, Fahal A, et al (2007) Melanin biosynthesis in Madurella mycetomatis and its effect on susceptibility to itraconazole and ketoconazole. Microbes Infect 9: 1114–1123. 17644456 10.1016/j.micinf.2007.05.015

[6] Visse R, Nagase H (2003) Matrix metalloproteinases and tissue inhibitors of metalloproteinases: structure, function, and biochemistry. Circ Res 92: 827–839. 12730128 10.1161/01.RES.0000070112.80711.3D

[7] Bauvois B (2012) New facets of matrix metalloproteinases MMP-2 and MMP-9 as cell surface transducers: outside-in signaling and relationship to tumor progression. Biochim Biophys Acta 1825: 29–36. 22020293 10.1016/j.bbcan.2011.10.001

[8] Ro Y, Hamada C, Inaba M, Io H, Kaneko K, et al (2007) Inhibitory effects of matrix metalloproteinase inhibitor ONO-4817 on morphological alterations in chlorhexidine gluconate-induced peritoneal sclerosis rats. Nephrol Dial Transplant 22: 2838–2848. 17545675 10.1093/ndt/gfm323

[9] Corbel M, Caulet-Maugendre S, Germain N, Molet S, Lagente V, et al (2001) Inhibition of bleomycin-induced pulmonary fibrosis in mice by the matrix metalloproteinase inhibitor batimastat. J Pathol 193: 538–545. 11276015 10.1002/path.826

[10] Lemaitre V, Kim HE, Forney-Prescott M, Okada Y, D'Armiento J (2009) Transgenic expression of matrix metalloproteinase-9 modulates collagen deposition in a mouse model of atherosclerosis. Atherosclerosis 205: 107–112. 19144335 10.1016/j.atherosclerosis.2008.11.030

[11] Vadla GP, Vellaichamy E (2012) Anti-fibrotic cardio protective efficacy of aminoguanidine against streptozotocin induced cardiac fibrosis and high glucose induced collagen up regulation in cardiac fibroblasts. Chem Biol Interact 197: 119–128. 22543014 10.1016/j.cbi.2012.04.005

[12] Hsu LS, Lee HH, Chen KM, Chou HL, Lai SC (2005) Matrix metalloproteinase-2 and -9 in the granulomatous fibrosis of rats infected with Angiostrongylus cantonensis. Ann Trop Med Parasitol 99: 61–70. 15701257 10.1179/136485905X19919

[13] Price SJ, Greaves DR, Watkins H (2001) Identification of novel, functional genetic variants in the human matrix metalloproteinase-2 gene: role of Sp1 in allele-specific transcriptional regulation. J Biol Chem 276: 7549–7558. 11114309 10.1074/jbc.M010242200

[14] Zhang B, Ye S, Herrmann SM, Eriksson P, de Maat M, et al (1999) Functional polymorphism in the regulatory region of gelatinase B gene in relation to severity of coronary atherosclerosis. Circulation 99: 1788–1794. 10199873 10.1161/01.cir.99.14.1788

[15] Meijer MJ, Mieremet-Ooms MA, van Hogezand RA, Lamers CB, Hommes DW, et al (2007) Role of matrix metalloproteinase, tissue inhibitor of metalloproteinase and tumor necrosis factor-alpha single nucleotide gene polymorphisms in inflammatory bowel disease. World J Gastroenterol 13: 2960–2966. 17589947 10.3748/wjg.v13.i21.2960 PMC4171149

[16] van de Sande WW, Fahal A, Tavakol M, van Belkum A (2010) Polymorphisms in catechol-O-methyltransferase and cytochrome p450 subfamily 19 genes predispose towards Madurella mycetomatis-induced mycetoma susceptibility. Med Mycol 48: 959–968. 20184498 10.3109/13693781003636680

[17] van de Sande WW, Fahal A, Verbrugh H, van Belkum A (2007) Polymorphisms in genes involved in innate immunity predispose toward mycetoma susceptibility. J Immunol 179: 3065–3074. 17709521 10.4049/jimmunol.179.5.3065

[18] Netti PA, Berk DA, Swartz MA, Grodzinsky AJ, Jain RK (2000) Role of extracellular matrix assembly in interstitial transport in solid tumors. Cancer Res 60: 2497–2503. 10811131

[19] Chakrabarti S, Patel KD (2005) Matrix metalloproteinase-2 (MMP-2) and MMP-9 in pulmonary pathology. Exp Lung Res 31: 599–621. 16019990 10.1080/019021490944232

[20] Borregaard N, Cowland JB (1997) Granules of the human neutrophilic polymorphonuclear leukocyte. Blood 89: 3503–3521. 9160655

[21] Van den Steen PE, Dubois B, Nelissen I, Rudd PM, Dwek RA, et al (2002) Biochemistry and molecular biology of gelatinase B or matrix metalloproteinase-9 (MMP-9). Crit Rev Biochem Mol Biol 37: 375–536. 12540195 10.1080/10409230290771546

[22] Trocme C, Gaudin P, Berthier S, Barro C, Zaoui P, et al (1998) Human B lymphocytes synthesize the 92-kDa gelatinase, matrix metalloproteinase-9. J Biol Chem 273: 20677–20684. 9685427 10.1074/jbc.273.32.20677

[23] Duncan ME, Richardson JP, Murray GI, Melvin WT, Fothergill JE (1998) Human matrix metalloproteinase-9: activation by limited trypsin treatment and generation of monoclonal antibodies specific for the activated form. Eur J Biochem 258: 37–43. 9851689 10.1046/j.1432-1327.1998.2580037.x

[24] Ferry G, Lonchampt M, Pennel L, de Nanteuil G, Canet E, et al (1997) Activation of MMP-9 by neutrophil elastase in an in vivo model of acute lung injury. FEBS Lett 402: 111–115. 9037177 10.1016/s0014-5793(96)01508-6

[25] Yazdan-Ashoori P, Liaw P, Toltl L, Webb B, Kilmer G, et al (2011) Elevated plasma matrix metalloproteinases and their tissue inhibitors in patients with severe sepsis. J Crit Care 26: 556–565. 21439766 10.1016/j.jcrc.2011.01.008

[26] Ahmed AO, Abugroun ES (1998) Unexpected high prevalence of secondary bacterial infection in patients with mycetoma. J Clin Microbiol 36: 850–851. 9508332 10.1128/jcm.36.3.850-851.1998 PMC104645

[27] van Hellemond JJ, Vonk AG, de Vogel C, Koelewijn R, Vaessen N, et al (2013) Association of eumycetoma and schistosomiasis. PLoS Negl Trop Dis 7: e2241. 23717704 10.1371/journal.pntd.0002241 PMC3662663

[28] Fujita H, Sakamoto N, Ishimatsu Y, Kakugawa T, Hara S, et al (2011) Effects of doxycycline on production of growth factors and matrix metalloproteinases in pulmonary fibrosis. Respiration 81: 420–430. 21502778 10.1159/000324080

